# The role of miR-155-5p in inflammation and mechanical loading during intervertebral disc degeneration

**DOI:** 10.1186/s12964-024-01803-7

**Published:** 2024-08-28

**Authors:** Petra Cazzanelli, Mikkael Lamoca, Johannes Hasler, Oliver Nic Hausmann, Addisu Mesfin, Varun Puvanesarajah, Wolfgang Hitzl, Karin Wuertz-Kozak

**Affiliations:** 1 Department of Biomedical Engineering, Rochester Institute of Technology, Rochester, NY USA; 2 Neuro- and Spine Center, Hirslanden Klinik St. Anna, Lucerne, Switzerland; 3 Neurosurgical Department, University of Berne, Berne, Switzerland; 4 Medstar Orthopaedic Institute, Georgetown University School of Medicine Washington, Washington, DC USA; 5 Department of Orthopedics and Rehabilitation, University of Rochester Medical Center, Rochester, NY USA; 6 Research and Innovation Management (RIM), Paracelsus Medical University, Salzburg, Austria; 7 Department of Ophthalmology and Optometry, Paracelsus Medical University, Salzburg, Austria; 8 Research Program Experimental Ophthalmology and Glaucoma Research, Paracelsus Medical University, Salzburg, Austria; 9 Schön Clinic Munich Harlaching, Spine Center, Academic Teaching Hospital and Spine Research Institute of the Paracelsus Medical University Salzburg (Austria), Munich, Germany

**Keywords:** MiRNA-155, Cyclic stretching, ECM degradation, MAPK signaling, Degenerative disc disease, Low back pain

## Abstract

**Background:**

Intervertebral disc (IVD) degeneration is a multifactorial pathological process resulting in the dysregulation of IVD cell activity. The catabolic shift observed in IVD cells during degeneration leads to increased inflammation, extracellular matrix (ECM) degradation, aberrant intracellular signaling and cell loss. Importantly, these pathological processes are known to be interconnected and to collectively contribute to the progression of the disease. MicroRNAs (miRNAs) are known as strong post-transcriptional regulators, targeting multiple genes simultaneously and regulating numerous intracellular pathways. Specifically, miR-155-5p has been of particular interest since it is known as a pro-inflammatory mediator and contributing factor to diseases like cancer and osteoarthritis. This study investigated the role of miR-155-5p in IVD degeneration with a specific focus on inflammation and mechanosensing.

**Methods:**

Gain- and loss-of-function studies were performed through transfection of human Nucleus pulposus (NP) and Annulus fibrosus (AF) cells isolated from degenerated IVDs with miR-155-5p mimics, inhibitors or their corresponding non-targeting control. Transfected cells were then subjected to an inflammatory environment or mechanical loading. Conditioned media and cell lysates were collected for phosphorylation and cytokine secretion arrays as well as gene expression analysis.

**Results:**

Increased expression of miR-155-5p in AF cells resulted in significant upregulation of interleukin (IL)-8 cytokine secretion during cyclic stretching and a similar trend in IL-6 secretion during inflammation. Furthermore, miR-155-5p mimics increased the expression of the brain-derived neurotrophic factor (BDNF) in AF cells undergoing cyclic stretching. In NP cells, miR-155-5p gain-of-function resulted in the activation of the mitogen-activated protein kinase (MAPK) signaling pathway through increased phosphorylation of p38 and p53. Lastly, miR-155-5p inhibition caused a significant increase in the anti-inflammatory cytokine IL-10 in AF cells and the tissue inhibitor of metalloproteinases (TIMP)-4 in NP cells respectively.

**Conclusion:**

Overall, these results show that miR-155-5p contributes to IVD degeneration by enhancing inflammation through pro-inflammatory cytokines and MAPK signaling, as well as by promoting the catabolic shift of AF cells during mechanical loading. The inhibition of miR-155-5p may constitute a potential therapeutic approach for IVD degeneration and low back pain.

**Supplementary Information:**

The online version contains supplementary material available at 10.1186/s12964-024-01803-7.

## Introduction

Intervertebral disc (IVD) degeneration causes the deterioration of the connective tissue located between vertebrae. One of its key characteristics is the multifactorial dysregulation of IVD cell activity through a catabolic shift resulting in extracellular matrix (ECM) degradation, inflammation, apoptosis, and senescence, as well as nerve and blood vessel ingrowth [1–3]. These processes are known to be highly interconnected, each of them contributing to the enhancement of the other degenerative processes, creating feedforward loops that collectively drive the progression of the disease [4].

Focusing on the deterioration of the IVD tissue, a disruption of metabolic homeostasis is observed, where native cells switch from ECM synthesis (anabolism) to ECM degradation (catabolism). Specifically, the secretion of matrix metalloproteinases (MMPs) is increased and the expression of ECM components like collagen and proteoglycans is reduced [5, 6]. Furthermore, the number of functional cells in the tissue decreases during degeneration due to the above-mentioned apoptosis and senescence which contributes to reduced capabilities of the tissue to produce ECM [7–9]. This disruption of tissue homeostasis changes the IVD’s composition and ultrastructure and can cause loss of disc height, dehydration of the nucleus pulposus (NP), fissures in the annulus fibrosus (AF), endplate defects, and in worst-case scenarios, disc bulging and herniation [3, 10, 11]. Apart from these macroscopic structural changes caused by ECM degradation, which also contribute to aberrant mechanical loading and altered biomechanics, the avascular nature of the tissue and the catabolic shift of IVD cells lead to the accumulation of fragmented, degraded ECM molecules [1, 4]. These ECM fragments are known to induce changes in the expression profile of IVD cells through pattern recognition receptors such as toll-like receptor (TLR)-2 and TLR-4 [12]. Upon activation and downstream signaling, these receptors lead to the increased secretion of pro-inflammatory cytokines, chemokines, and proteases, hence establishing a link between inflammation and ECM degradation [13, 14].

Inflammation is one of the key processes driving IVD degeneration from the onset of the pathology to its advanced stages. The changes in cell activity of native cells lead to increased secretion of pro-inflammatory and pro-catabolic cytokines, such as interleukins (IL-1β, IL-6, IL-8, IL-17), tumor necrosis factor (TNF), and chemokine (C‑C) ligands (CCL-2) [15, 16]. Furthermore, the inflammatory environment activates intracellular signaling pathways mitogen-activated protein kinase (MAPK) and nuclear factor NF-κB through phosphorylation of their key enzymes extracellular signal-regulated kinases (ERKs), p38, and c-Jun N-terminal kinases (JNKs) [17]. Downstream effects of MAPK and NF-κB signaling are apoptosis [18], senescence [19], enhanced MMP expression [20], and secretion of pro-inflammatory cytokines [21, 22]. The increased accumulation of pro-inflammatory cytokines and neurotrophic factors, accompanied by the disruption of tissue integrity, can lead to the infiltration of immune cells and innervation. Recruited immune cells contribute to degeneration and the inflammatory environment by increasing chemokine and cytokine release and leading to sensitization of nerve roots, resulting in nociception and discogenic pain [23–25]. This is one of the major distinguishing factors between asymptomatic and symptomatic IVD degeneration [24, 25]. Symptomatic IVD degeneration, commonly termed degenerative disc disease (DDD), is one of the major contributing factors of low back pain (LBP), the leading cause of years lived in disability worldwide that poses a significant socioeconomic burden [26, 27].

Due to the important role of the IVD tissue in spinal kinematics and the structural and biomechanical changes occurring during degeneration, an understanding of the impact of mechanical loads on cell activity is crucial. Non-physiological mechanical stress is known to induce catabolic and inflammatory cascades, matrix degradation, and senescence [28–30]. Multiple cell receptors, channels, and signaling pathways are involved in the transduction of aberrant mechanical loading, including MAPK, ERK, and transient receptor potential channels [31, 32]. Hyperphysiological mechanical loading has been shown to further IVD degeneration. Cyclic stretching of AF cells at high strain of 8–20% induces downregulation of anabolic factors (ACAN, COL2) [33] and upregulation of catabolic and inflammatory factors (MMP1, MMP3, MMP9, MMP13, IL-1β, IL-6, IL-8, TLR-2, TNF, NGF) [33–35]. Therefore, mechanical stress and its impact on IVD cell activity is an essential factor in the degenerative process and its multifactorial nature.

As strong post-transcriptional regulators, miRNAs are known for targeting numerous genes and cell mechanisms and are hence of specific importance in multifactorial pathologies. These small non-coding RNAs regulate gene expression by binding to the 3’-untranslated region (3’-UTR) of target mRNAs leading to their reduced expression or degradation [36]. It is well known that miRNAs are pleiotropic and can affect cell behavior through the modulation of signaling pathways, especially during miRNA dysregulation in pathologies [37–39]. Amongst miRNAs characterized in IVD degeneration [39], miR-155-5p is of specific interest. Previous research of our group linked increased TLR-2 signaling to upregulation of miR-155-5p in degenerated and non-degenerated human IVD cells [40]. Furthermore, multiple studies in different tissues suggest that miR-155-5p plays an important pro-inflammatory role in pathologies like cancer [41], arthritis [42], and neuroinflammatory disorders [43]. In studies conducted with cells of the immune system (macrophages and dendritic cells), upregulation of miR-155-5p through TLR-2 and TLR-4 enhanced the cell’s inflammatory response in vitro and *in vivo* [44–47]. Furthermore, miR-155-5p was upregulated in innate immune cells and microglia under neuroinflammatory conditions [48] and its inhibition offered mild protection against retinal degeneration by reducing inflammation [49]. Additionally, miR-155-5p overexpression in human osteoarthritis knee cartilage was shown to suppress autophagy in chondrocytes [50]. MiRNAs have also been extensively investigated as biomarkers in numerous pathologies and a recent study showed that miR-155-5p levels in patient serum might be a possible biomarker for lumbar DDD [51]. However, the effects of miR-155-5p have so far not been studied in the broader context of IVD degeneration.

In this study, we provide a comprehensive overview of the role of miR-155-5p in intervertebral disc degeneration with a specific focus on its effects on IVD cell activity during inflammation and mechanosensing. To that end, we investigate changes in the expression and secretion of the key catabolic factors (matrix-degrading enzymes, innervation factors, pro-inflammatory cytokines and chemokines) and intracellular signaling following miR-155-5p gain- or loss-of-function in an inflammatory environment and during mechanical loading.

## Materials and methods

### Human IVD cell isolation and culture

Biopsies from patients undergoing spinal surgery due to disc herniation or DDD were obtained for in vitro experiments with human degenerated IVD cells. As described previously, NP, AF or mixed IVD tissue was excised intraoperatively, diced and digested enzymatically overnight with 0.2% collagenase NB4 (Nordmark) and 0.3% dispase II (Sigma-Aldrich) in 1 × Dulbecco's Phosphate Buffered Saline (DPBS, Cytiva) with 5% antibiotic–antimycotic (anti-anti, Gibco) at 37 °C, 5% CO_2_ [31]. After enzymatic tissue digestion, cells were kept in culture up to passages 1–2 in growth medium consisting of Dulbecco’s Modified Eagle’s Medium/Ham's F-12 medium (DMEM/F-12, Cytiva) supplemented with 10% fetal bovine serum (FBS, Cytiva) and 1% anti-anti. An overview of the patients characteristics can be found in Supplementary Table 1.

### Transfection of miRNA mimics/inhibitors

In order to transfect human IVD cells with miRNAs, 3 uL HiPerFect Transfection Reagent (Qiagen, 301705) were mixed and incubated with 50 nM miRNA mimics/inhibitors or their corresponding non-targeting controls for 10 min at room temperature to allow for the formation of transfection complexes (hsa-miR-155-5p miRCURY LNA miRNA Mimic YM00472490-ADA, Qiagen, 339173; hsa-miR-155-5p miRCURY LNA miRNA Power Inhibitor YI04101510-DDA, Qiagen, 339131; negative control miRCURY LNA miRNA Mimic YM00479902-ADB, Qiagen, 339173; negative control B miRCURY LNA miRNA Power Inhibitor YI00199007-DDA, Qiagen, 339136). Human IVD cells were then reverse-transfected at a density of 20,000 cells/cm^2^ with miR-155-5p mimic/inhibitor or non-targeting complexes in no-serum media (DMEM/F12 with 0.1% anti-anti). After 24 h the medium was changed to growth medium in order to let the cells recover. The efficiency of the miRNA mimic/inhibitor transfection was analyzed by reverse transcription-quantitative polymerase chain reaction (RT-qPCR) 72 h post transfection.

### Induction of inflammation in IVD cells

Effects of miRNA gain- and loss-of-function on the inflammatory cell response were studied by first transfecting human degenerated IVD cells with miRNA mimics or inhibitors as described above. After 22 h of recovery in growth medium, cells were starved for 2 h in no-serum medium followed by treatment with 5 ng/mL recombinant human IL-1β (PeproTech, 200-01B). For the analysis of protein phosphorylation, cells were treated with 5 ng/mL IL-1β for 30 min before being lysed for protein extraction. Secretion of cytokines and catabolic factors as well as gene expression were analyzed after treating AF and NP cells for 24 h with 5 ng/mL IL-1β. To that end, cell culture supernatants were collected for cytokine/MMP arrays and IVD cells were lysed for miRNA and mRNA extraction 72 h post transfection.

### Fabrication and characterization of stretching chambers

Polydimethylsiloxane (PDMS) stretching chambers were fabricated using a 1:1 mixture of Sylgard 184 (Dow, 2646340) and Sylgard 527 Silicone Dielectric Gel Clear 0.9 kg Kit (Dow, 1,696,742), cast using an aluminum mold with dimensions fitting the automated cell stretching system and cured at room temperature overnight, at 80 °C for 40 min or at 140 °C for 15 min. After demolding, the stretching chambers were cleaned in 70% ethanol for 5 min in an ultrasound bath, rinsed with deionized water, dried and plasma-treated with the plasma cleaner PDC-001 (Harrick Plasma) before being used for characterization or cyclic stretching.

The stiffness of the stretching chambers was determined by performing tensile testing with the UniVert tensile tester (CellScale Biomaterials Testing) at a crosshead speed of 10 mm/s until the point of failure using a 1–10 N and 10–100 N load cell. The Young’s Modulus was calculated using the stress/strain curve.

Digital image correlation (DIC) was used to determine the difference between engineering strain and true strain of the fabricated PDMS chambers during cyclic sinusoidal uniaxial loading with the automated cell stretching system (STB-140–10, STREX). To that end, fluorescent ink (Millennium Colorworks, Ink Glow UV) was incorporated into the PDMS mixture before casting and curing the chambers at 140 °C for 15 min. The stretching chambers were then imaged during cyclic sinusoidal uniaxial loading with the StrainMaster Portable System (LaVision) at an image sampling rate of 100 Hz using blue LED illumination. Calibration, data collection, image processing, and data analysis were performed using DaVis Software 10.2.1 (LaVision).

### Cyclic stretching

Sterile PDMS stretching chambers were coated overnight with 50 μg/mL fibronectin (EMD Millipore, FC010) at 37 °C. The following day, human IVD cells, with or without simultaneous reverse-transfection of miRNA mimics/inhibitors, were seeded in the stretching chambers (20,000 cells/cm^2^, 10 cm^2^ cell culture surface). After the transfection and recovery phase (46 h post transfection), cells were starved in no-serum media for 2 h and then subjected to 8% cyclic sinusoidal uniaxial engineering strain for 24 h at a frequency of 1 Hz at 37 °C and 5% CO_2_. Control chambers were kept in identical conditions without stretching. Cell lysates and conditioned media were harvested for expression analysis, cytokine and MMP arrays 72 h post transfection (experimental timeline depicted in Supplementary Fig. 1).

### miRNA extraction and RT-qPCR

MiRNA extraction for the expression analysis was performed with the miRNeasy Tissue/Cells Advanced Mini Kit (Qiagen, 217604). Following IL-1β treatment or mechanical loading, cells were rinsed twice with ice-cold 1 × DPBS, lysed with 260 uL lysis buffer, and the samples were further processed according to the manufacturer’s instructions. The concentration and quality of miRNA were analyzed with the UV–Vis spectrophotometer NanoPhotometer N50 (Implen). Reverse transcription of miRNAs was achieved with the miRCURY LNA RT Kit (Qiagen, 339340) according to the manufacturer’s protocol using 20 ng total RNA per 20 μL reaction followed by quantitative PCR with the miRCURY LNA SYBR Green PCR Kit (Qiagen, 339347). The respective miRCURY LNA miRNA PCR Assays were used for the analysis of the expression of specific miRNAs (YP00204308—hsa-miR-155-5p, YP00204063—hsa-miR-103a-3p, Qiagen, 339306). Furthermore, spike-ins were used to monitor the consistency of the extraction process and reverse transcription (RNA Spike-In Kit, Qiagen, 339390). The results are shown as 2^−∆∆Ct^ values relative to the housekeeping miRNA miR-103a-3p and control conditions (cells transfected with non-targeting miRNA mimics or inhibitors).

### Cytokine and MMP array

In order to analyze the secretion of cytokines and MMPs, conditioned media was collected immediately after treatment and centrifuged at 500 g for 5 min for the removal of cell debris. The resulting supernatants were used undiluted for the Human Cytokine Array GS1 (RayBiotech Life, GSH-CYT-1–4) and Human MMP Array GS1 (RayBiotech Life, GSH-MMP-1–4), which were performed according to the manufacturer’s instructions. The concentration range of the cytokine and MMP array proteins was detected at a range of 1 pg/mL – 1 ng/mL. The median fluorescent signal was normalized to the plate background and array’s positive control. Fold changes of fluorescence were calculated relative to the non-targeting control conditions.

### Phosphorylation array

The phosphorylation of proteins associated with the MAPK pathway was determined with the Human/Mouse MAPK Phosphorylation Array (RayBiotech Life, AAH-MAPK-1–8). To that end, cells were rinsed with ice-cold 1 × DPBS and lysed on ice with 60 µL radioimmunoprecipitation assay (RIPA) buffer supplemented with 100 × phosphatase and protease inhibitor cocktail, which was diluted to a final concentration of 1 × in RIPA buffer (Thermo Fisher Scientific, 89,900 and 78,440). The lysate was incubated for 30 min at 4 °C with gently shaking followed by centrifugation at 14,000 g for 10 min at 4 °C. Protein concentrations were quantified with the BCA protein assay (Thermo Fisher Scientific, 23,225). The samples were then diluted to a final concentration of 75 µg/mL in RIPA buffer containing phosphatase and protease inhibitor cocktail and 1 mL of the diluted samples was used for the MAPK Phosphorylation Array, according to the manufacturer’s instructions. The membranes were scanned with the chemiluminescence imaging system Odyssey XF imaging system (LI-COR Biosciences) and analyzed with the Empiria Studio Software (LI-COR Biosciences). Signal intensities were normalized to the positive control and membrane background. The results are shown as fold changes relative to the non-targeting controls.

### RNA extraction and gene expression analysis (RT-qPCR)

Analysis of gene expression was performed by co-extracting mRNA with the miRNeasy Tissue/Cells Advanced Mini Kit (Qiagen, 217604) according to the manufacturer’s recommendations. After determining the quantity and quality of RNA with the NanoPhotometer N50 (Implen), 500–1000 ng RNA were used for reverse transcription into cDNA with the High-Capacity cDNA Reverse Transcription Kit with RNase Inhibitor (Thermo Fisher Scientific, 4374967). Finally, gene expression was quantified by qPCR with the TaqMan Fast Advanced Master Mix (Thermo Fisher Scientific, 4444963) and the respective Taqman Gene Expression Assay (Hs00153936_m1 ACAN,

Hs00171458_m1 NGF, Hs02718934_s1 BDNF, Hs00174103_m1 CXCL8/IL-8, Hs00153133_m1 PTGS2/COX2, Hs00427620_m1 TBP, Thermo Fisher Scientific, 4331182) using the QuantStudio™ 3 (Thermo Fisher Scientific). The results were calculated as 2^−∆∆Ct^ values relative to the housekeeping gene TBP and control conditions.

### Cell viability assay

The effect of PDMS on cell viability was determined with the alamarBlue assay. Cells were seeded on PDMS stretching chambers and kept in culture for 72 h. Thereafter, cells were incubated with a 1:10 dilution of alamarBlue Cell Viability Reagent (Thermo Fisher Scientific, DAL1025) for 4 h at 37 °C. Cells seeded in standard 6-well tissue culture plates were used as a positive control and cells lysed in a 6-well tissue culture plate were used as a negative control. Furthermore, the cytotoxicity of cyclic stretching was tested using the CyQUANT™ LDH Cytotoxicity Assay Kit (Thermo Fisher Scientific, C20301) following the manufacturer’s instructions. Briefly, conditioned media of cells undergoing cyclic stretching for 24 h was collected and 50 µL of each sample was used for the LDH cytotoxicity assay. Non-stretched cells were used as a positive control and lysed cells were used as a negative control.

### Statistical analysis

To check normality, Saphiro-Wilks test was used and Levene’s test to test variance homogeneity. Bootstrap-t tests with and without the assumption of variance homogeneity were used to test means. 95% bias-corrected and accelerated (BCa) confidence intervals were computed to estimate the difference of means. All reported tests were two-sided, and *p*-values < 0.05 were considered statistically significant. All statistical analyses in this report were performed by use of NCSS (NCSS 10, NCSS, LLC. Kaysville, UT), and Wolfram Research, Inc., Mathematica, Version 13.1, Champaign, IL (2022).

## Results

### Effects of miR-155-5p expression on cytokine secretion during inflammation

**Fig. 1:**
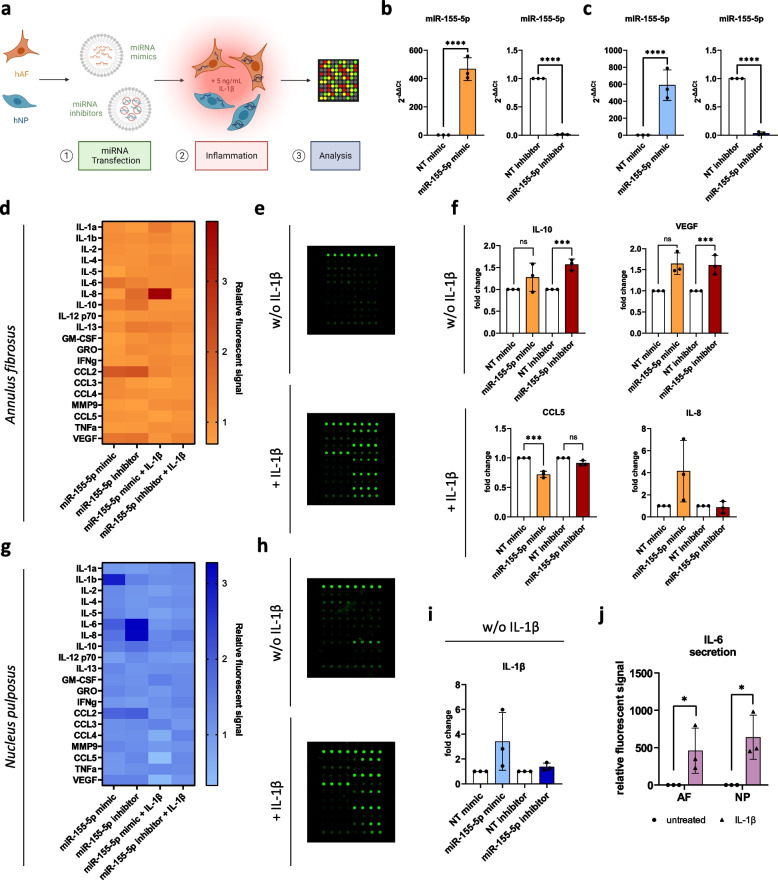
The effect of miR-155-5p gain-/loss-of-function on cytokine secretion. **a** Schematic of the experimental setup using human degenerated AF and NP cells. **b-c** Expression analysis of miR-155-5p in AF (**b**) and NP (**c**) cells following transfection with mimics, inhibitors or non-targeting (NT) controls. **d-e** Human cytokine secretion array performed with AF cells transfected with miR-155-5p mimics or inhibitors, untreated (w/o IL-1β) or being subjected to IL-1β treatment (+ IL-1β); heatmap of the fluorescent signal relative to non-targeting controls (**d**) and arrays scanned with a fluorescent laser scanner (**e**). **f** Changes in secretion of IL-10, VEGF, CCL5 and IL-8 in AF cells. **g-h** Human cytokine secretion array performed with AF cells transfected with miR-155-5p mimics or inhibitors, untreated (w/o IL-1β) or being subjected to IL-1β treatment (+ IL-1β); heatmap of the fluorescent signal relative to non-targeting controls (**g**) and arrays scanned with a fluorescent laser scanner (**h**). **i** Changes in secretion of IL-1β in NP cells. j Secretion of IL-6 following treatment with IL-1β compared to untreated AF and NP cells. (*n* = 3), mean ± SD, ns = not significant, * *p* < 0.05, *** *p* < 0.001, **** *p* < 0.0001

Pro-inflammatory cytokines and chemokines are known key mediators that drive multiple pathological processes during IVD degeneration, including ECM degradation, inflammation and apoptosis [15]. Furthermore, higher levels of cytokines like TNF, IL-6 and IL-8 are linked to painful degenerative disk disease [24] and can cause the infiltration of host immune cells as well as increased sensitization of nerve fibers upon AF and cartilage endplate ruptures [52]. Since miR-155-5p is largely attributed to being a pro-inflammatory miRNA [42, 53], the effect of miR-155-5p gain- and loss-of-function on the secretion of cytokines was studied. To that end, AF and NP cells were transfected with miR-155-5p mimics or inhibitors and then exposed to an inflammatory environment by treatment with IL-1β (Fig. 1a). Changes in miR-155-5p expression were confirmed in AF and NP cells, with increases of 466.5 ± 80.6 fold change (*p* < 0.0001, AF cells) and 588.9 ± 179.9 fold change (*p* < 0.0001, NP cells) following transfection with miRNA mimics and decreases of 0.01 ± 0.006 fold change (*p* < 0.0001, AF cells) and 0.03 ± 0.025 (*p* < 0.0001, NP cells) following transfection with miRNA inhibitors (Fig. 1b, c). Quantification of cytokine secretion of AF cells (Fig. 1d, e) showed that the inhibition of miR-155-5p resulted in a significantly increased secretion of anti-inflammatory cytokine IL-10 (1.6 ± 0.1 relative fluorescent signal, *p* < 0.001), while miR-155-5p under IL-1β treatment let to a decrease of CCL5 (0.7 ± 0.1 relative fluorescent signal, *p* < 0.001) and to a recognizable though not significant increase of IL-8 (4.1 ± 2.8 relative fluorescent signal, *p* = 0.129) due to high donor-donor variability (Fig. 1f). The vascular endothelial growth factor (VEGF) was significantly increased upon miR-155-5p inhibition (1.6 ± 0.2 relative fluorescent signal, *p* < 0.001) and interestingly miR-155-5p mimics also showed an increase, even though not significant. The transfection of NP cells with miR-155-5p mimics resulted in a clear increase of IL-1β secretion (3.4 ± 2.3 relative fluorescent signal, *p* = 0.242) even though not significant (Fig. 1g-i). The induction of inflammation in AF and NP cells after treatment with IL-1β was confirmed by detecting a significant increase in IL-6 secretion compared to untreated cells, though high variability in inflammatory response was observed between donors (Fig. 1j). Overall these results suggest that miR-155-5p might play a pro-inflammatory role in IVD degeneration.

### Role of miR-155-5p in ECM degradation and innervation

**Fig. 2:**
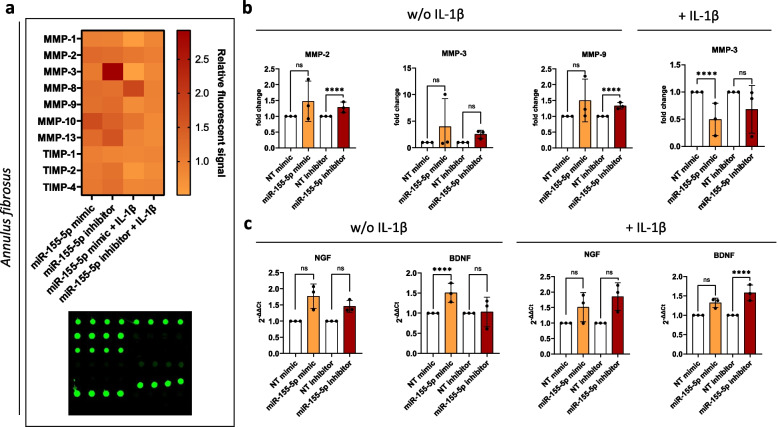
ECM degradation and innervation under miR-155-5p gain-/loss-of-function. **a** Human MMP secretion array performed with AF cells transfected with miR-155-5p mimics or inhibitors, untreated (w/o IL-1β) or being subjected to IL-1β treatment (+ IL-1β); heatmap of the fluorescent signal relative to non-targeting controls and array scanned with a fluorescent laser scanner. **b** Changes in the secretion of MMP-2, -3, and -9. **c** Gene expression of NGF and BDNF. (*n* = 3), mean ± SD, ns = not significant, **** *p* < 0.0001

**Fig. 3:**
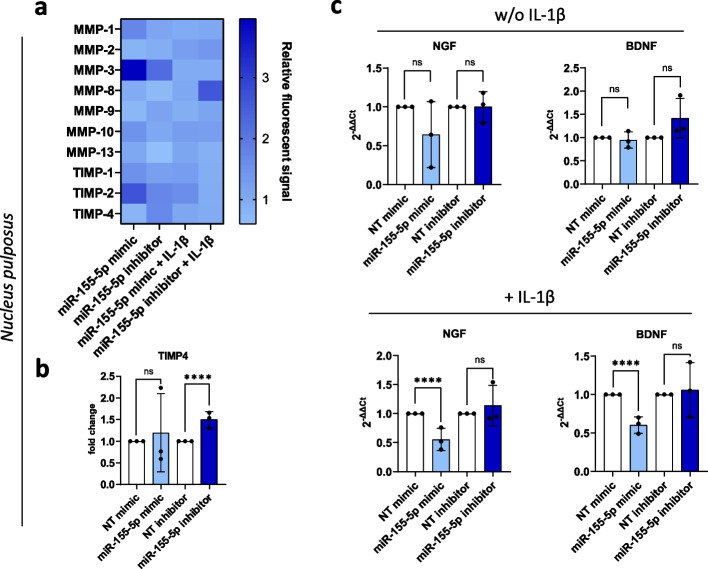
ECM degradation and innervation under miR-155-5p gain-/loss-of-function. **a** Human MMP secretion array performed with NP cells transfected with miR-155-5p mimics or inhibitors, untreated (w/o IL-1β) or being subjected to IL-1β treatment (+ IL-1β); heatmap of the fluorescent signal relative to non-targeting controls and array scanned with a fluorescent laser scanner. **b** Changes in the secretion of MMP-2, -3, and -9. **c** Gene expression of NGF and BDNF. (*n* = 3), mean ± SD, ns = not significant, **** *p* < 0.000

Degradation of the ECM in IVD tissue occurs due to the increased secretion of ECM-degrading enzymes and the decreased capability of IVD cells to produce ECM components like aggrecan and collagen [6, 54]. This leads to structural and molecular changes in the ECM as well as to the accumulation of ECM fragments and the disruption of tissue integrity and biomechanics [5]. Consequently, immune cells and nerve fibers are able to infiltrate the tissue. The ingrowth of nerve fibers is enhanced further by the secretion of nerve growth factor (NGF) and brain-derived neurotrophic factor (BDNF) by IVD cells [55]. Since we observed increases in some pro-inflammatory cytokines caused by miR-155-5p expression and due to the close link between inflammation and ECM degradation, the effect of miR-155-5p expression on MMPs and their inhibitors (tissue inhibitor of metalloproteinases, TIMPs) was studied (Fig. 2a and Fig. 3a). AF cells showed significant increases in MMP-2 (1.28 ± 0.17 relative fluorescent signal, *p* < 0.0001) and MMP-9 (1.33 ± 0.10 relative fluorescent signal, *p* < 0.0001) when transfected with miR inhibitors without IL-1β treatment and a similar trend in MMP-3 (2.55 ± 0.78 relative fluorescent signal, *p* < 0.0001). In contrast, mimics of miR-155-5p decreased MMP-3 secretion (0.5 ± 0.3 relative fluorescent signal, *p* < 0.0001) under an inflammatory environment (Fig. 2b). In NP cells, inhibition of miR-155-5p caused a significant increase in the MMP inhibitor TIMP4 (1.5 ± 0.2 relative fluorescent signal, *p* < 0.0001) (Fig. 3b).

Furthermore, the expression of NGF, BDNF and aggrecan were analyzed following the gain- or loss-of-function of miR-155-5p. While no significant changes in aggrecan (ACAN) were observed (Supplementary Fig. 2), miR-155-5p mimics caused a significant increase in BDNF expression (1.51 ± 0.23 fold change, *p* < 0.0001) and a similar trend in NGF expression under non-inflammatory conditions (1.77 ± 0.38 fold change, *p* = 0.141) in AF cells (Fig. 2c). Treatment with IL-1β led to a significant increase in BDNF expression following miR-155-5p inhibition (1.58 ± 0.21 fold change, *p* < 0.0001)(Fig. 2c). In contrast, miR-155-5p mimics caused a significant downregulation of NGF (0.55 ± 0.19 fold change, *p* < 0.0001) and BDNF (0.61 ± 0.10 fold change, *p* < 0.0001) under inflammatory conditions and a similar trend in untreated NP cells (Fig. 3c).

These results show that the effect of miR-155-5p on ECM degradation and innervation is distinctively different in AF cells compared to NP cells. While miR-155-5p leads to a general increase in MMPs and neurotrophic factors in AF cells, downregulation of neurotrophic factors is observed in NP cells (Table 1). Furthermore, some of these effects are reversed when inflammation is induced by IL-1β.

**Table 1:** Inflammation, ECM degradation and innervation under miR-155-5p gain-/loss-of-function

Cell Type	MiRNA Transfection	Inflammation	Gene Expression
AF cells	miR-155-5p mimics	w/o IL-1β	BDNF^a^ ↑ NGF^b^ ↑
		+ IL-1β	CCL5^a^ ↓ IL-8^b^ ↑ MMP-3^a^ ↓
	miR-155-5p inhibitor	w/o IL-1β	IL-10^a^ ↑ VEGF^a^ ↑ MMP-2^a^ ↑ MMP-9^a^ ↑
		+ IL-1β	BDNF^a^ ↑ NGF^a^ ↑
NP cells	miR-155-5p mimics	w/o IL-1β	IL-1β^b^ ↑ NGF^b^ ↓
		+ IL-1β	NGF^a^ ↓ BDNF^a^ ↓
	miR-155-5p inhibitor	w/o IL-1β	TIMP4^a^ ↑
		+ IL-1β	n/a

Changes in gene expression in AF and NP cells transfected with miR-155-5p mimics or inhibitors, untreated (w/o IL-1β) or being subjected to IL-1β treatment (+ IL-1β). Significant results are marked with ^a^, while evident though not statistically significant trends are marked with ^b^

### Regulation of MAPK signaling pathway through miR-155-5p

**Fig. 4:**
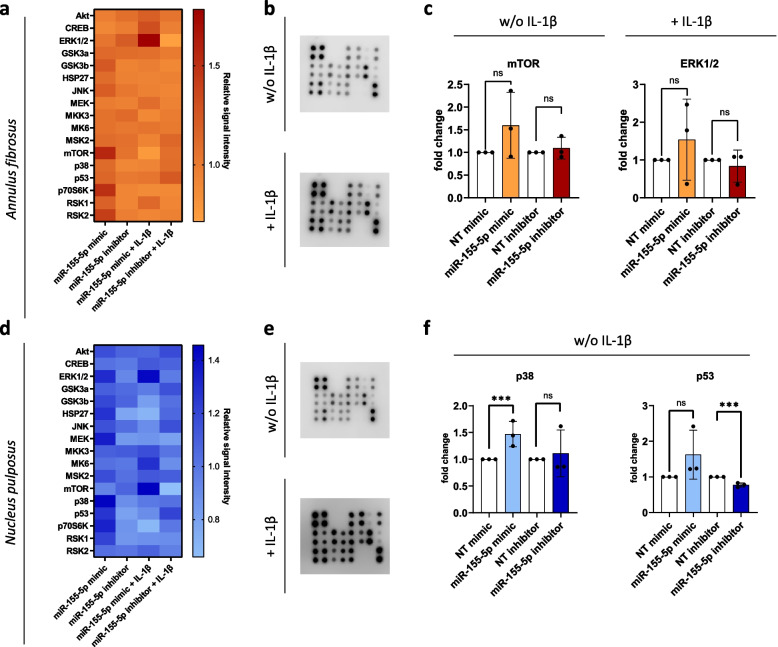
The effect of miR-155-5p gain-/loss-of-function on MAPK signaling. **a-d** Heatmap of MAPK phosphorylation array performed with AF (**a**) and NP (**d**) cells transfected with miR-155-5p mimics or inhibitors, untreated (w/o IL-1β) or being subjected to IL-1β treatment (+ IL-1β). **b-e** MAPK arrays of AF (**b**) and NP (**e**) cells scanned with a chemiluminescent imaging system (**e**). **c** Changes in phosphorylation of mTOR and ERK1/2 in AF cells. **f** Changes in phosphorylation of p38 and p53 in NP cells. (*n* = 3), mean ± SD, ns = not significant, *** *p* < 0.001

Cellular stresses like inflammation, hyperosmotic stress or mechanical loading are transmitted through the intracellular signaling pathways such as MAPK, NF-kB, and PI3/Akt, which are known to be dysregulated in IVD degeneration [4]. Three major subfamilies of the MAPK pathway are prevalent in IVD degeneration: ERK, JNK and p38. These subfamilies can be activated through different stimuli and lead to distinctive cell responses. Furthermore, extensive crosstalk between MAPK and other intracellular pathways like NF-κB, AKT, and mTOR can occur [4]. In order to determine whether the above-seen increases in pro-inflammatory cytokines and other catabolic factors are mediated through MAPK, we studied the effect of miR-155-5p expression on MAPK pathway phosphorylation in AF (Fig. 4a) and NP cells (Fig. 4d). Moreover, inflammation was induced to study changes in MAPK signaling upon its activation through IL-1β (Fig. 4b,e). AF cells showed an apparent though not significant increase in mTOR phosphorylation (1.60 ± 0.73 relative signal intensity, *p* = 0.25) upon transfection with miR-155-5p mimics and a similar trend under inflammation in ERK1/2 phosphorylation (1.54 ± 1.07 relative signal intensity, *p* = 0.36) (Fig. 4c). In NP cells, miR-155-5p expression led to a significant increase of p38 phosphorylation (1.49 ± 0.23 relative signal intensity, *p* < 0.001) and miR-155-5p inhibition decreased p53 phosphorylation (0.73 ± 0.01 relative signal intensity, *p* < 0.001) (Fig. 4f). This indicates that miR-155-5p enhances MAPK signaling, possibly regulating cell activity through different MAPK subfamilies in AF and NP cells and hence leading to distinct downstream cell responses.

### Fabrication and characterization of stretching chambers for uniaxial cyclic stretching

**Fig. 5:**
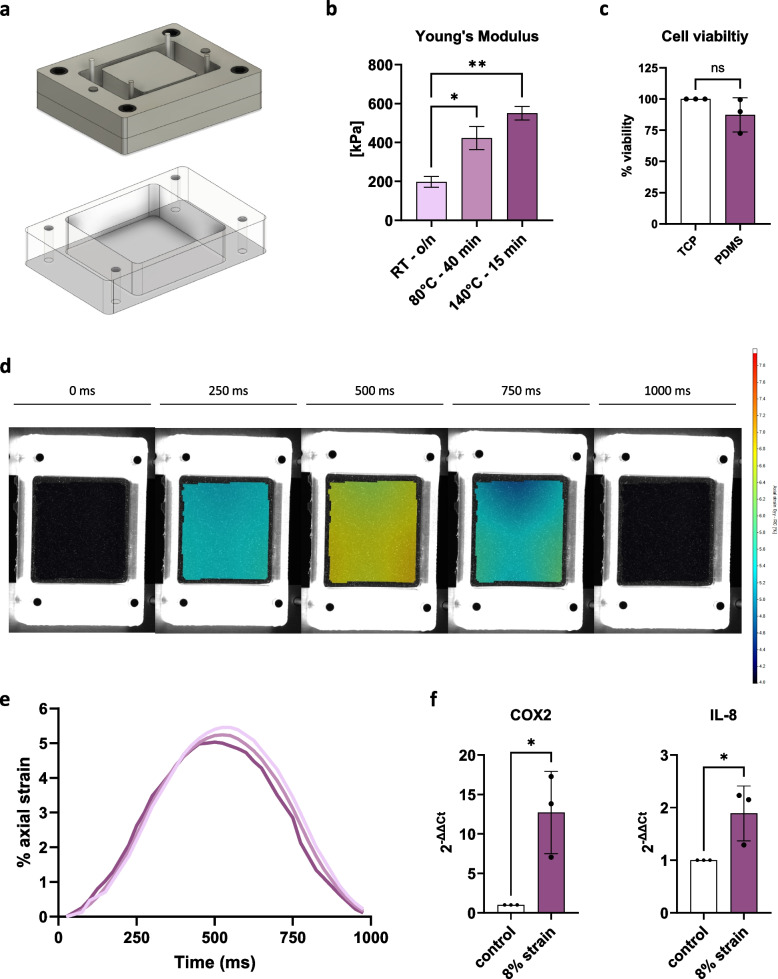
Characterization of PDMS stretching chambers and cyclic stretching conditions. **a** Schematic of aluminum mold and PDMS stretching chambers. **b** Young’s modulus of stretching chambers undergoing different curing conditions (RT o/n: room temperature overnight) **c** Cell viability of AF cells cultured on PDMS chambers cured at 140 °C for 15 min. **d**-**e** Axial strain distribution images (**d**) and distribution curve (**e**). **f** Gene expression of COX2 and IL-8. (*n* = 3), mean ± SD, ns = not significant, * *p* < 0.05, ** *p* < 0.01

Mechanical stimuli are known to be beneficial for IVD homeostasis at physiological levels. However, non-physiological mechanical loading can contribute to apoptosis, catabolism and inflammation [56]. Furthermore, structural changes during degeneration such as reduction in disc height, increased tissue stiffness and loss of water-binding proteoglycans result in aberrant mechanical loading and reduced capability of the tissue to bear compressive loads [3, 29, 57]. In order to study the effects of miR-155-5p expression and inhibition on mechanosensing, PDMS stretching chambers were fabricated for uniaxial cyclic stretching using an aluminum mold fitting the dimensions of the STREX automated cell stretching system (Fig. 5a). To simulate the stiffness of degenerated AF tissue, which has previously been reported to be 567 ± 7 kPa [58], different curing conditions were tested, followed by tensile testing for the investigation of the Young’s modulus. While lower curing temperatures and longer curing intervals produced chambers with lower stiffnesses (Fig. 5b), curing PDMS at 140 °C for 15 min resulted in a Young’s modulus coherent with the one of degraded AF tissue [58]. Following the determination of optimal curing conditions, cell viability was tested and no impact on cell viability was seen in cells cultured on PDMS chambers compared to standard tissue culture plates (87.3 ± 13.6% cell viability) (Fig. 5c). Furthermore, the true strain during cyclic stretching was determined through DIC, showing that while applying 8% engineering strain at 1 Hz frequency, PDMS stretching chambers were undergoing an axial deformation of 5.2 ± 0.2% (Fig. 5d, e) and a transverse deformation of 1.69 ± 0.1% (Supplementary Fig. 3). The difference between engineering strain to true strain is caused by reduced deformation of PDMS chambers due to the selected higher stiffness. Lastly, cell responses to cyclic stretching with 8% engineering strain at 1 Hz were tested and significant increases in the expression of pro-inflammatory cytokines COX-2 (12.7 ± 5.2 fold change, *p* < 0.05) and IL-8 (1.89 ± 0.5 fold change, *p* < 0.05) were observed (Fig. 5f).

### The effect of miR-155-5p expression on cytokine secretion during cyclic stretching

**Fig. 6:**
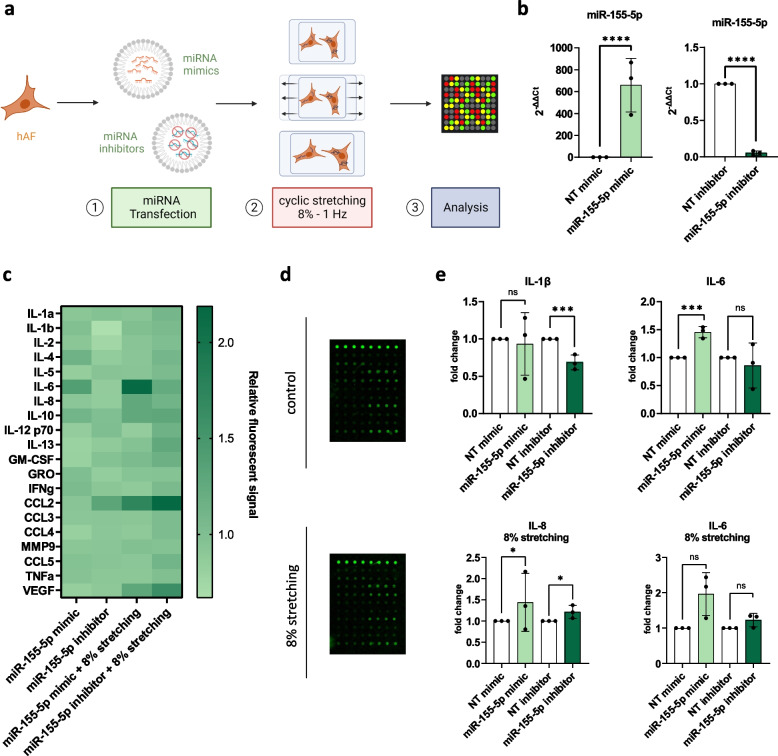
The effect of miR-155-5p gain-/loss-of-function on cytokine secretion during cyclic stretching. **a** Schematic of the experimental setup. **b** Expression analysis of miR-155-5p following transfection with mimics, inhibitors or non-targeting (NT) controls. **c**-**d** Human cytokine secretion array performed with AF cells transfected with miR-155-5p mimics or inhibitors, being subjected to cyclic stretching (8% strain); heatmap of the fluorescent signal relative to non-targeting controls (**c**) and arrays scanned with a fluorescent laser scanner (**d**). **e** Changes in secretion of IL-1β, IL-6 and IL-8. (*n* = 3), mean ± SD, ns = not significant, * *p* < 0.05, *** *p* < 0.001, **** *p* < 0.0001

Due to the known contributing effect of mechanical loading to degeneration through the downregulation of anabolic markers and upregulation of catabolic markers as well as pro-inflammatory mediators [34, 59], the effects of miR-155-5p gain-/loss-of-function on cytokine secretion during mechanical loading was studied. To that end, AF cells were seeded in PDMS stretching chambers and transfected with miR mimics and inhibitors (Fig. 6a). The increase and decrease of miR-155-5p expression following transfection was confirmed by RT-qPCR (659.2 ± 245.6 fold change, *p* < 0.0001; 0.05 ± 0.03 fold change, *p* < 0.0001) (Fig. 6b). The cytokine secretion array showed that inhibition of miR-155-5p reduced the secretion of IL-1β (0.6 ± 0.1 relative fluorescent signal, *p* < 0.001) and miR-155-5p mimics caused a significant increase in IL-6 (1.5 ± 0.1 relative fluorescent signal, *p* < 0.001) (Fig. 6d, e). During cyclic stretching, a significant increase was seen in IL-8 upon transfection with miR-155-5p mimic (1.4 ± 0.4 relative fluorescent signal, *p* < 0.05), while its inhibitor showed a similar but reduced increase (1.2 ± 0.2 relative fluorescent signal, *p* < 0.05). Furthermore, an apparent though not significant increase in IL-6 by miR-155-5p mimic was observed during cyclic stretching (1.9 ± 0.6 relative fluorescent signal, *p* = 0.1) (Fig. 6e). This shows that miR-155-5p expression causes increases in the pro-inflammatory cytokines IL-6 and IL-8, while its inhibition can reduce the secretion of IL-1β.

### Role of miR-155-5p in catabolism during mechanical loading

**Fig. 7:**
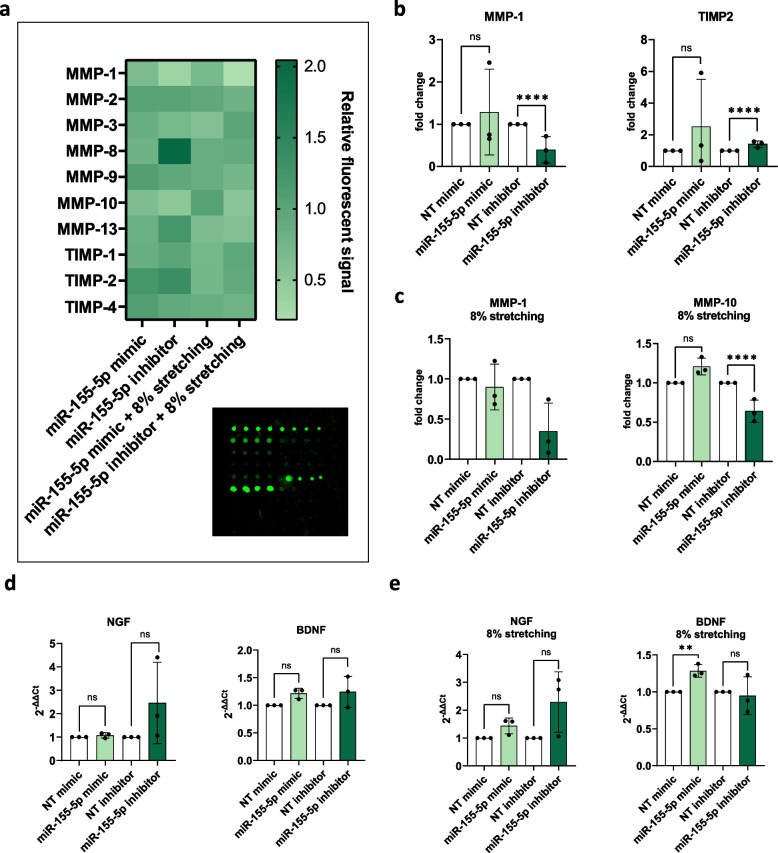
ECM degradation and innervation under miR-155-5p gain-/loss-of-function and cyclic loading. **a** Human MMP secretion array performed with AF cells transfected with miR-155-5p mimics or inhibitors, being subjected to cyclic stretching (8% strain); heatmap of the fluorescent signal relative to non-targeting controls and array scanned with a fluorescent laser scanner. **b** Changes in the secretion of MMP-1, MMP-10, and TIMP2. **c** Gene expression of NGF and BDNF. (*n* = 3), mean ± SD, ns = not significant, ** *p* < 0.01, **** *p* < 0.0001

Lastly, the role of miR-155-5p expression on catabolic factors like ECM-degrading proteins (MMPs) and neurotrophic factors (NGF, BDNF) as well as anabolic factors (ACAN) was tested (Fig. 7a). The inhibition of miR-155-5p caused the decreased secretion of MMP-1 (0.4 ± 0.3 relative fluorescent signal, *p* < 0.0001), while the secretion of the MMP inhibitor TIMP2 was increased (1.4 ± 0.2 relative fluorescent signal, *p* < 0.0001) (Fig. 7b). While undergoing mechanical loading, inhibition of miR-155-5p furthermore caused significant downregulation of MMP-10 (0.6 ± 0.1 relative fluorescent signal, *p* < 0.0001) and a similar, though not significant trend in MMP-1. When studying the expression of neurotrophic factors, miR-155-5p expression caused a significant increase of BDNF under cyclic stretching (1.28 ± 0.1 fold change, *p* < 0.001), while inhibition showed an apparent though not significant trend in upregulating NGF. No significant changes in aggrecan expression were observed (Supplementary Fig. 4). Taking these results together, miR-155-5p seems to promote the catabolic shift in AF cells during mechanical loading (Table 2).

**Table 2:** Inflammation, ECM degradation and innervation under miR-155-5p gain-/loss-of-function during mechanical loading

Cell Type	MiRNA Transfection	Inflammation	Gene Expression
AF cells	miR-155-5p mimics	w/o strain	IL-6^a^ ↑
		8% strain	IL-8^a^ ↑ IL-6^b^ ↑ BDNF^a^ ↑
	miR-155-5p inhibitor	w/o strain	IL-1β^a^ ↓ MMP-1^a^ ↓ TIMP2^a^ ↑ NGF^b^ ↑
		8% strain	MMP-10^a^ ↓ MMP-1^b^ ↓ NGF^b^ ↑

Changes in gene expression in AF cells transfected with miR-155-5p mimics or inhibitors, untreated (w/o strain) or being subjected to cyclic stretching (8% strain). Significant results are marked with ^a^, while evident though not statistically significant trends are marked with ^b^

## Discussion

**Fig. 8:**
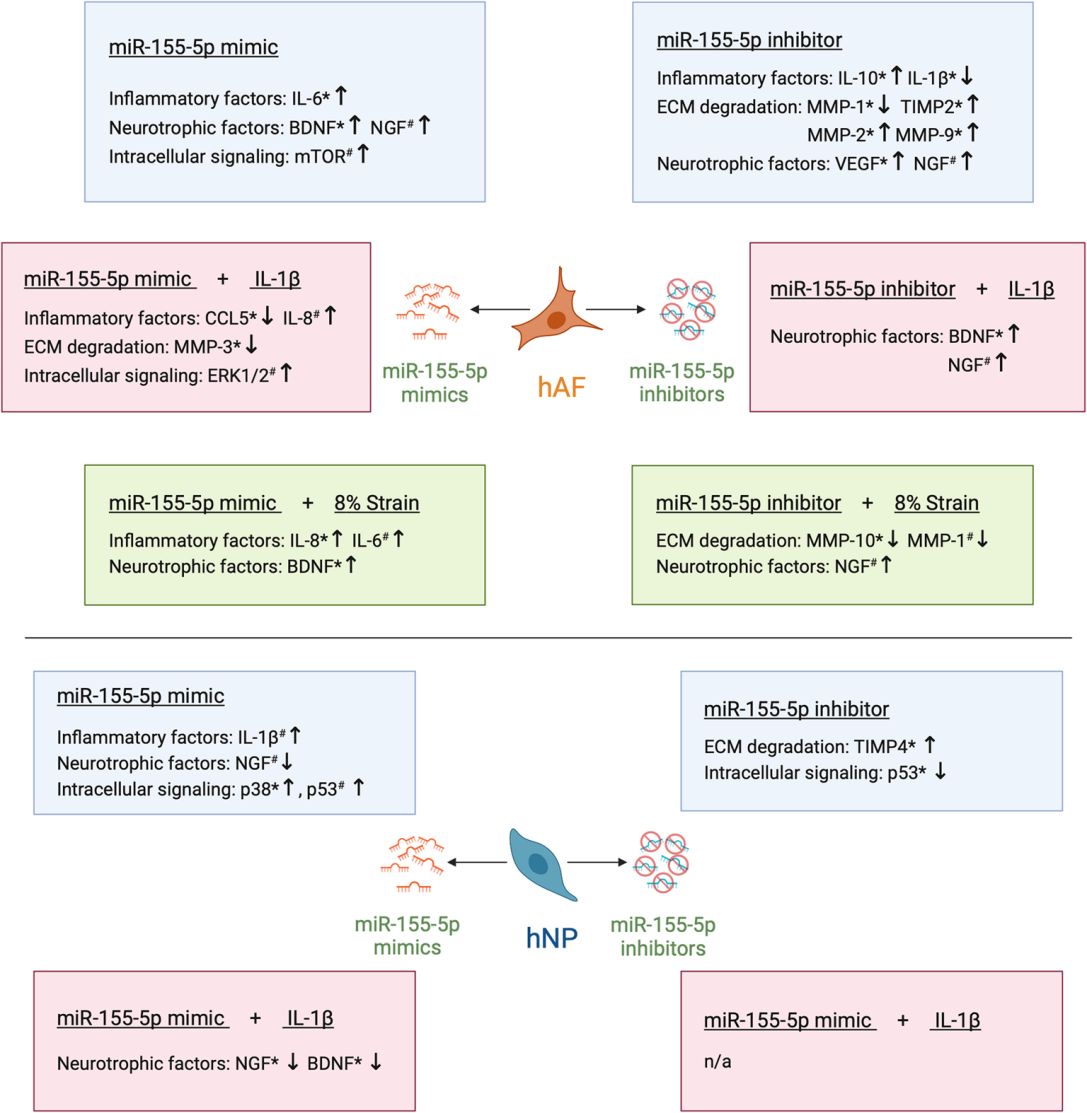
The role of miR-155-5p gain-/loss-of-function in degenerated hAF and hNP cells during inflammation (IL-1ß treatment), mechanical loading (8% strain) or without additional stimuli. The results show the effects of miR-155-5p mimics and inhibitors on inflammatory factors, neurotrophic factors, ECM degradation and intracellular signaling (upregulation shown as ↑, downregulation shown as ↓). Significant results are marked with *, while evident though not statistically significant trends are marked with #

The multifactorial dysregulation of cell activity during IVD degeneration poses a major challenge in understanding the underlying pathological processes and their etiology, but also in the development of therapeutics. The interplay between the key biological processes driving degeneration further amplifies the complexity of the pathology [4]. Therefore, the study of miRNAs has become of increasing interest in recent years, due to their capability to regulate intracellular processes and target multiple genes simultaneously [39]. Furthermore, miRNAs are known to be involved in numerous pathologies [41–43] and more recent research has also shown that miRNAs dysregulation can be affected by mechanical forces, hence being mechanosensitive [60]. The involvement of miR-155-5p in neoplastic and inflammatory diseases has been studied extensively in recent years, with its expression frequently being upregulated under pathological conditions [61]. Our group has previously reported that miR-155-5p is upregulated in IVD cells following TLR-2 activation and miR-155-5p target prediction identified multiple targets in relevant pathways and transcription factors like cFOS [40]. The goal of the current study was to gather a comprehensive analysis of the role of miR-155-5p in IVD degeneration, with a specific focus on inflammation, ECM degradation, MAPK signaling, and mechanical loading (Fig. 8).

The occurrence of chronic inflammation during IVD degeneration is often termed the distinguishing factor between asymptomatic IVD degeneration and symptomatic degenerative disc disease [15]. MiR-155-5p has been identified as a pro-inflammatory mediator in other tissues and pathologies such as neuroinflammation [43], fibrosis [62, 63], and arthritis [42]. Moreover, miR-155-5p is a known regulator of the immune system [47]. When studying the role of miR-155-5p in inflammation of the IVD, we found that the inhibition of miR-155-5p increased the secretion of the anti-inflammatory cytokine IL-10 as well as VEGF in AF cells. Furthermore, our data show apparent trends of miR-155-5p mimics increasing IL-8 secretion in AF cells under inflammatory conditions and IL-1β in NP cells under non-inflammatory conditions. Similarly, miR-155-5p inhibition in AF cells cultured in PDMS stretching chambers reduced IL-1β secretion and miR-155-5p mimics increased IL-6 and IL-8 secretion during cyclic stretching. These results indicate that miR-155-5p plays a pro-inflammatory role in IVD cells during inflammation and mechanosensing. This finding is in concordance with studies in arthritis, where miR-155-5p drives inflammatory activation of macrophages and monocytes by targeting inhibitors of TLR and cytokine receptor pathways, thus resulting in increased production of the cytokines TNF, IL-6, IL-8, and IL-1β [64–66]. Regarding the role of miR-155-5p during mechanical loading, very few studies have investigated this interplay so far. The results of a study conducted with endothelial cells suggest that miR-155-5p might be involved in altering RhoA and the actin cytoskeleton organization during unidirectional shear stress [67]. Importantly, mechanical stimuli affecting AF cells within the IVD are of a highly complex nature. While cyclic stretching in 2-dimensional culture allows for investigation of the role of miR-155-5p in a comprehensive manner affecting the cells through axial and transverse strains, it would be essential to study these effects further, accounting for the range of strain and shear stresses.

The role of miR-155-5p in ECM degradation and innervation was studied with a specific focus on the secretion of MMPs and gene expression of the neurotrophic factors NGF and BDNF in AF and NP cells. These studies showed highly tissue-specific changes following miR-155-5p gain- or loss-of-function, with distinct differences between AF and NP cells. We found that NP cells show an increase in the MMP inhibitor TIMP4 following the inhibition of miR-155-5p. These results suggest that miR-155-5p inhibition in NP cells might reduce matrix degradation. In AF cells, miR-155-5p inhibition resulted in minor upregulation of MMPs under non-inflammatory conditions and in downregulation of MMPs during cyclic stretching. Under inflammatory conditions, miR-155-5p mimics reduced MMP-3 secretion. These results show not only tissue-specific changes but also distinct effects of miR-155-5p gain- or loss-of-function in AF cells depending on the type of external stressors such as inflammation or mechanical loading. However, the interaction of miR-155-5p and MMP secretion has not been thoroughly studied in IVD cells so far and future investigations should provide a better understanding of the effect of miR-155-5p on ECM degradation. This would be of particular interest since studies in chondrocytes have shown similar results, where upregulation of miR-155-5p led to the increase of MMP-3 and MMP-13 during inflammation [68]. The effect of miR-155-5p on the expression pattern of NGF and BDNF showed also highly tissue-specific changes. In NP cells miR-155-5p downregulated both NGF and BDNF. On the other hand, AF cells showed an upregulation of BDNF with miR-155-5p mimics under non-inflammatory conditions and during cyclic stretching, while during IL-1β treatment upregulation of BDNF was facilitated by miR-155-5p inhibition. This shows a similar cell stress-dependent response to miR-155-5p gain- or loss-of-function in AF cells, as well as a general increase of neurotropic factors in AF cells. These results should be further investigated with ex vivo and in vivo studies, since recent publications have shown that miR-155-5p promoted dorsal root ganglion neuron axon growth [69], while miR-155-5p inhibition reduced neuropathic pain [70] and promoted spinal cord repair [71].

Numerous intracellular pathways are involved in the transduction of external stressors to the downstream dysregulation of gene expression during IVD degeneration. The MAPK signaling pathway is known to be a key regulator of catabolism and inflammation in IVD tissue. Importantly, distinct genes are upregulated depending on which subfamily of MAPK signaling proteins are activated [4]. When studying the effect of miR-155-5p on the MAPK signaling pathway, NP cells showed increased phosphorylation of p38 by miR-155-5p mimics and a reduction in p53 phosphorylation by miR-155-5p inhibitors. Since it is known that activation of p38 results in increased expression of pro-inflammatory cytokines and ECM-degrading enzymes, these results suggest that increased miR-155-5p expression might contribute to IVD degeneration by regulating p38 signaling in NP cells. Due to the detrimental role of p38 in NP cells during IVD degeneration, its inhibition has been discussed as a potential therapeutic strategy [72]. Furthermore, the downregulation of p53 by miR-155-5p inhibition could suggest a possible involvement of miR-155-5p in cell fate, which should be investigated in further studies. Gain- and loss-of-function studies of miR-155-5p in AF cells showed trends of increased mTOR phosphorylation by mimics under non-inflammatory conditions, while ERK1/2 was activated under inflammatory conditions. These results demonstrate the tissue-specific activation of distinct MAPK signaling enzymes, likely causing distinct downstream effects in the catabolic regulation of NP and AF cells. Moreover, the interaction between miR-155-5p and MAPK signaling regulation has been recently studied in vivo showing that miR-155-5p inhibition reduced pain through SOCS1 and p38 [70]. In order to assess the effect of miR-155-5p and its inhibition on the progression of IVD degeneration and low back pain, future studies will examine selected downstream effects identified in this study within a larger donor cohort. This would allow for a more detailed examination of the specific miR-155-5p effects based on donor characteristics (age, degree of degeneration, pain, etc.), which was not feasible in this comprehensive study allowing only for a limited number of donors to be tested. Additionally, investigating the impact of miR-155-5p gain- and loss-of-function in healthy cells would be valuable for discerning the function of miR-155-5p during the onset of inflammation and degeneration, as opposed to its role under already prevalent inflammatory and degenerative conditions.

## Conclusion

In conclusion, this study gives a comprehensive overview of the role of miR-155-5p in IVD pathophysiology. We demonstrate that miR-155-5p enhances MAPK signaling and inflammation in NP cells and contributes to the catabolic shift during cyclic stretching of AF cells. Future studies will focus on the effects of miR-155-5p in IVD degeneration in vivo, testing the therapeutical potential of its inhibition for the reduction of inflammation and discogenic low back pain.

### Supplementary Information

Supplementary Material 1.

## Data Availability

The datasets generated and/or analyzed during the current study are available in the Figshare repository, 10.6084/m9.figshare.24712842.
