# Supplementary material for: The role of miR-155-5p in inflammation and mechanical loading during intervertebral disc degeneration
Source: Cell Commun Signal. 2024 Aug 28;22:419. doi: 10.1186/s12964-024-01803-7 (PMC11351051; doi:10.1186/s12964-024-01803-7)
Supplement: Supplementary file 1 — Supplementary Material 1. [file 12964_2024_1803_MOESM1_ESM.docx]

# The Role of miR-155-5p in Inflammation and Mechanical Loading during Intervertebral Disc Degeneration

Petra Cazzanelli^1^, Mikkael Lamoca^1^, Johannes Hasler^1^, Oliver Nic Hausmann^2,3^, Addisu Mesfin^4^, Varun Puvanesarajah^5^, Wolfgang Hitzl^6,7,8^ and Karin Wuertz-Kozak^1,9^*

^1^ Department of Biomedical Engineering, Rochester Institute of Technology (RIT), Rochester, NY, USA

^2^ Neuro- and Spine Center, Hirslanden Klinik St. Anna, Lucerne, Switzerland

^3^ Neurosurgical Department, University of Berne, Berne, Switzerland

^4^ Medstar Orthopaedic Institute, Georgetown University School of Medicine Washington, DC, USA

^5^ Department of Orthopedics and Rehabilitation, University of Rochester Medical Center, Rochester, NY, USA

^6^ Research and Innovation Management (RIM), Paracelsus Medical University, Salzburg, Austria

^7^ Department of Ophthalmology and Optometry, Paracelsus Medical University, Salzburg, Austria

^8^ Research Program Experimental Ophthalmology and Glaucoma Research, Paracelsus Medical University, Salzburg, Austria

^9^ Schön Clinic Munich Harlaching, Spine Center, Academic Teaching Hospital and Spine Research Institute of the Paracelsus Medical University Salzburg (Austria), Munich, Germany

***Corresponding Author**

Karin Wuertz-Kozak

Department of Biomedical Engineering

Rochester Institute of Technology

160 Lomb Memorial Drive, Bldg. 73

Rochester, NY 14623 (USA)

kwbme@rit.edu

# Supplementary Material

**Supplementary Table 1**: Patient characteristics of surgical degenerated IVD biopsies. f = female; m = male; DDD = degenerative disc disease; L = lumbar; C = cervical.

| Donor | Age | Sex | Pfirrman Grade | Disc Level | Diagnosis |
| --- | --- | --- | --- | --- | --- |
| 1 | 72 | f | V | L5/S1 | DDD |
| 2 | 52 | f | IV | C5/6 | Herniation |
| 3 | 53 | m | III | C6/7 | Herniation |
| 4 | 55 | f | III | L5/S1 | DDD |
| 5 | 68 | f | IV | L5/S1 | Herniation |
| 6 | 60 | m | IV | L4/5 | Herniation |

**
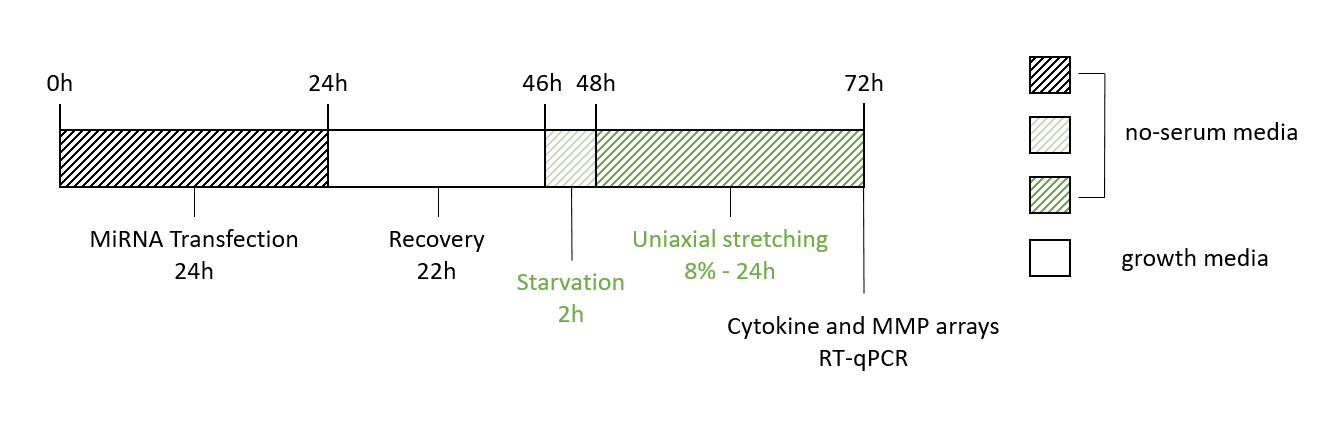
**

**Supplementary Figure 1** Experimental setup of *in vitro* cyclic stretching studies. Timeline of miRNA transfection, recovery phase and cyclic stretching.


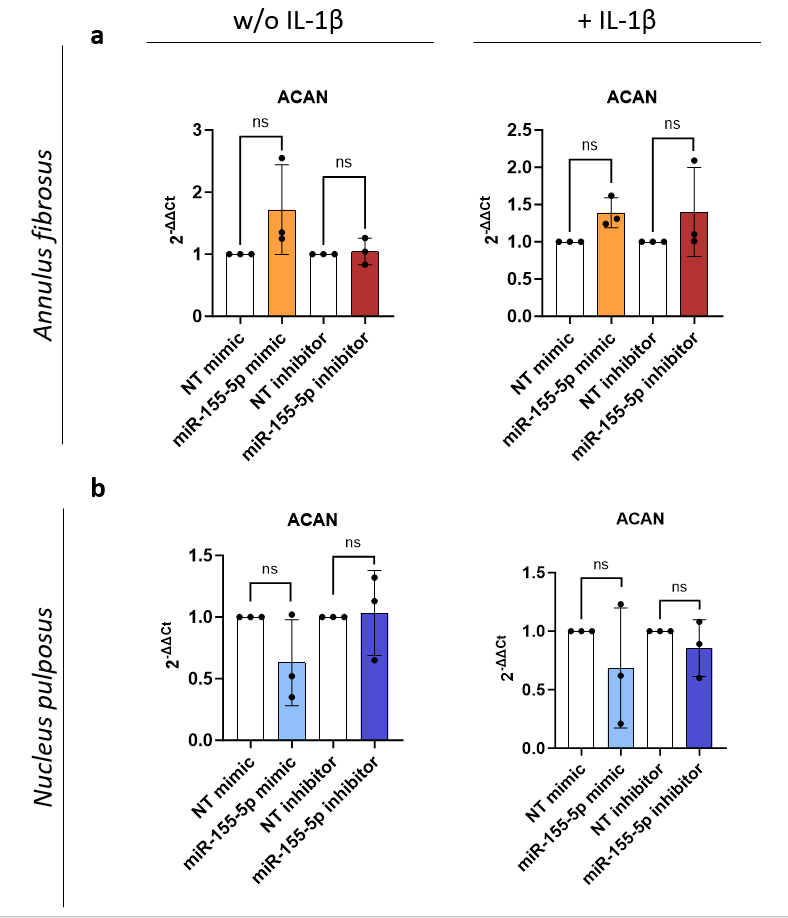


**Supplementary Figure 2** Gene expression analysis of Aggrecan (ACAN) in **a** AF and **b** NP cells transfected with miR-155-5p mimics or inhibitors, untreated (w/o IL-1β) or being subjected to IL-1β treatment (+IL-1β). (*n* =3), mean ± SD, ns = not significant


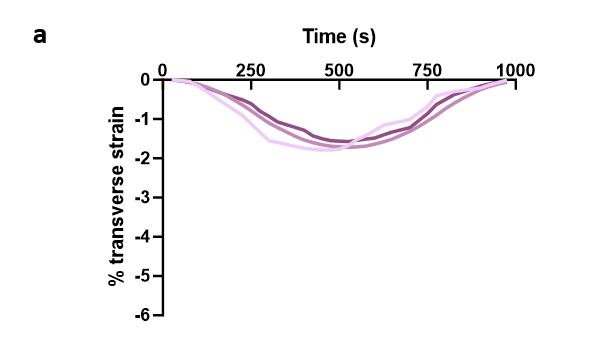


**Supplementary Figure 3** Characterization of PDMS stretching chambers during cyclic stretching conditions with DCI, showing the transverse strain distribution curve.

**
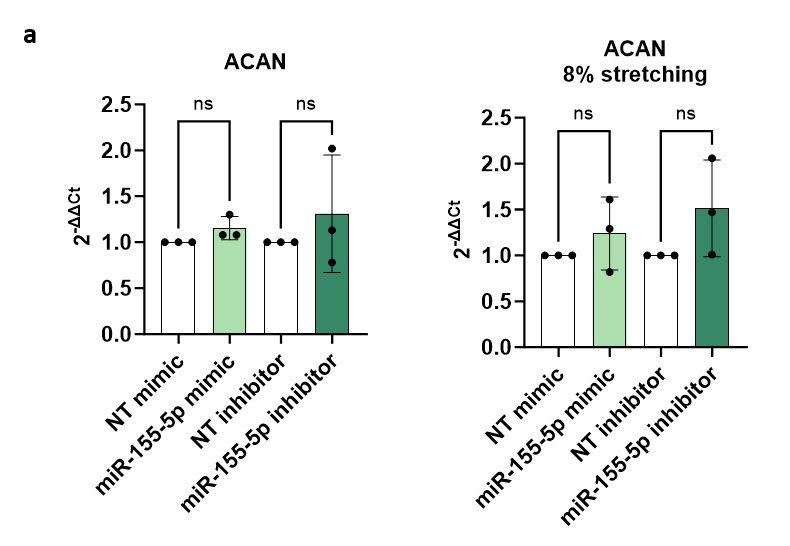
**

**Supplementary Figure 4** Gene expression analysis of Aggrecan (ACAN) in AF cells transfected with miR-155-5p mimics or inhibitors, untreated or being subjected to 8% strain. (*n* =3), mean ± SD, ns = not significant
